# Virus in the pathogenesis of inflammatory bowel disease: role of Toll-like receptor 7/8/3 

**Published:** 2021

**Authors:** Hamid Asadzadeh Aghdaei, Negar Jamshidi, Vahid Chaleshi, Nazanin Jamshidi, Amir Sadeghi, Mohsen Norouzinia, Mohammad Reza Zali

**Affiliations:** 1 *Basic and Molecular Epidemiology of Gastrointestinal Disorders Research Center, Research Institute for Gastroenterology and Liver Diseases, Shahid Beheshti University of Medical Sciences, Tehran, Iran*; 2 *Gastroenterology and Liver Diseases Research Center, Research Institute for Gastroenterology and Liver Diseases, Shahid Beheshti University of Medical Sciences, Tehran, Iran*

**Keywords:** Inflammatory bowel disease, COVID-19, toll-like receptors, Ulcerative colitis, Crohn’s disease

## Abstract

The pathogenesis of inflammatory bowel disease (IBD) is influenced by immune system malfunction, particularly innate immune receptors such as toll-like receptors. Furthermore, it is critical to investigate the extremely close association between viruses and IBD incidence. Toll-like receptors (TLRs) 3, 5, and 7 are involved in antiviral immune responses. Finding a relationship between TLR-related virus and IBD is important not only for understanding the disease pathogenesis, but also for developing effective therapies. It has been shown that influenza is expressed more severely in patients with IBD who use immune system inhibitors, and the influenza vaccine is less effective in these patients. In dendritic cells, TLR7 and TLR8 regulate the production of interferons (IFNs) and inflammatory mediators. COVID-19 causes the production of IL-6, possibly due to the induction of TLR pathways. TLR activation by SARS-CoV-2 causes inflammation and IL-1 production, which induces the production of IL-6. Understanding TLR-associated viruses’ molecular mechanisms can greatly help improve the quality of life of people with IBD. Therefore, the present study reviewed the role of TLR7, 8, and 3 in inflammatory bowel disease as well as their association with viral infections and evaluated different antagonists for the treatment of IBD.

## Introduction

 The two main types of inflammatory bowel disease (IBD) are Crohn’s disease and ulcerative colitis. Although these two groups have similarities, each has its own characteristics and behavior (1). IBDs are chronic intestinal inflammatory disorders diagnosed by distinct methods (2). Despite extensive studies on the environmental and infectious risk factors of the disease, the etiology of IBD is not entirely clear. It seems that genetic, environmental, and microbial factors affect this disease, and a higher prevalence is observed in some families and ethnicities (3, 4). 

Cellular receptors are one of the factors that help the innate immune system recognize infections and trigger immune responses. Toll-like receptors (TLRs) are members of the innate immunity receptor family, and they play a key role in cytokine modulation, adaptive immune system indirect activation, and detection of pathogen-associated molecular patterns (5). Various polymorphisms and mutations on these receptors have recently been shown to be linked to IBD (6). Moreover, the impact of viral infections in IBD has just lately been discovered (7, 8). Examining the pathways linked with viral infections might thus be extremely beneficial in the treatment of patients suffering from this condition. Moreover, numerous studies have shown that influenza presents more intensely in patients with IBD who consume immune system modulators, and the influenza vaccine is less effective in patients with IBD (9). There is concern that the frequency of Covid-19 infection in IBD patients is growing as a result of the usage of immunosuppressive medications (10). Antiviral innate immune responses are dependent on the TLR family of innate immune receptors. In fact, ssRNA and dsRNA viruses are detectable by TLR7/8 and TLR3, which produce antiviral immune responses by detecting viral infection, activating signaling pathways, and inducing the production of antiviral cytokines and chemokines. TLR7 and TLR8 control the production of interferons (IFNs) and inflammatory mediators in dendritic cells. TLR3 is one of the most essential TLRs for innate immune system activation (11, 12). However, a novel treatment strategy for IBD patients to enhance their quality of life may be developed by better understanding the biological processes of these viruses.

The goal of this review paper is to describe TLR pathways 3, 7, and 8 in connection with viral infections in IBD to get a better understanding of the role of intestinal viruses.

**Toll-like Receptors 7/8/3 Activation and Signaling**

**Figure 1:**
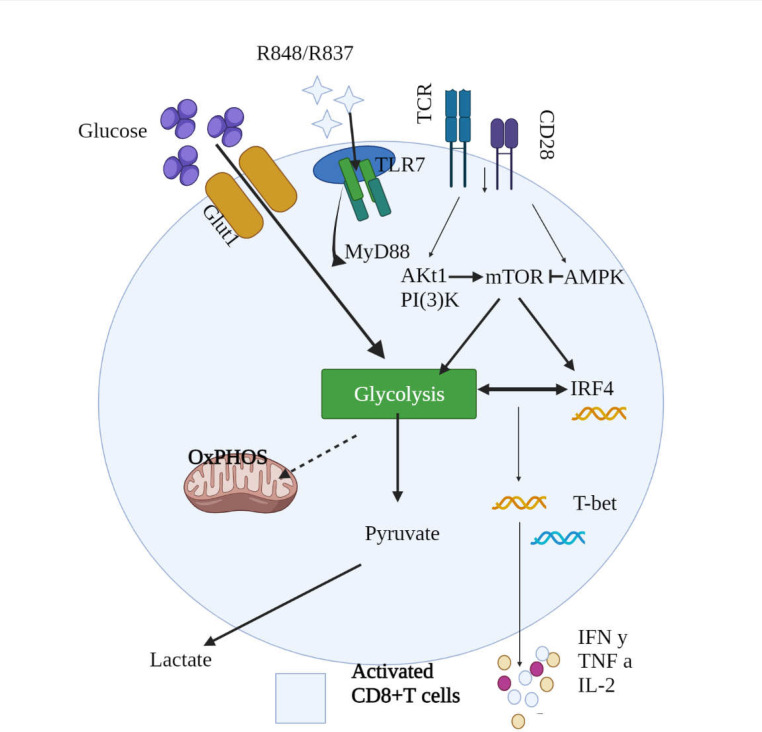
A schematic description of *TLR7*-mediated enhancement of CD8^+^ T cell function

TLRs act as a bridge between innate and acquired immunity following activation by ligands (13). They cause different antiviral responses, such as the release of inflammatory cytokines and maturation of antigen-presenting cells (APCs) (14-16). Potential mechanisms of TLR signaling pathways have two important downstream signaling cascades: A: differentiation primary response (MyD88), and B: IR-domain-containing adapter protein (TRIF/TICAM-1-) (17, 18). The MyD88-dependent pathways bind the MYD88 adapter protein to the Toll/IL-1 (TIR) receptor and invoke interleukin-1 (IL-1), receptor-associated kinase 4 (IRAK4), and TNF receptor-associated factor 6 (TRAF6). IRAK4 phosphorylates IRAK1 and eventually leads to the release and activation of NF-κB, which leads to transcription of proinflammatory cytokines such as interleukin 1β (IL-1β), tumor necrosis factor alpha (TNF-α), and interleukin 6 (IL-6) (18). The TRIF adaptor protein activates the TANK-binding kinase 1 (TBK1) and IKK pathways, resulting in the phosphorylation and activation of interferons (17). With the exception of TLR3, all TLRs signal through MYD88 cascades. Furthermore, the TLR signaling pathway is highly important in the control and clearance of viruses and contributes to the antiviral reaction by CD8^+^ T lymphocytes (CTLs) and the indirect activation of T and B lymphocytes (19). An investigation on a hepatitis B mouse model (HBV) demonstrated that the IL-1R/TLR signaling pathway is needed for the production of CD8^+^ T-cells (20). TLR7 ligands can improve acquired immune response and elevate APCs resulting in increased CD8^+^ T-cells (21, 22). Song et al. indicated the higher expression of TLR7 in CD8^+^ T-cells and augmented the expression of CD8^+^ T-cells activation markers through TLR7 stimulation in vitro (23). *TLR4*, *TLR5*, *TLR7*, and *TLR8* expression in host immunological responses CD8 ^+^ T cells during HIV-1 infection compared to healthy individuals was studied by Song et al. Their findings showed that only *TLR7* in T cells CD8 ^+ ^from HIV-1 infected persons had greater expression levels. Furthermore, HIV-1 infected individuals have aberrant *TLR7* expression in CD8^+ ^T cells, which may contribute to inappropriate immunological activation in HIV-1 infection and play a key role in the HIV-1 pathogenesis (23). Salerno et al. reported that the CD8^+^ T-cells of a mouse can be stimulated by TLR7 ligands, leading to the rapid production of IFN-γ (24). These findings showed that TLR7 can directly activate CD8^+^ T-cells and regulate their function. However, the fundamental mechanisms are not well known yet. According to Gang et al., TLR7 activates T-cells via the MyD88 signaling pathway, then MyD88 signaling enhances T-cell performance by activating the Akt- and protein kinase-dependent mTOR pathway (Figure 1) (25). Also, TLR7 induces CD8^+^ T-cells by way of P848. These cells are activated in case of expression by MyD88-dependent αCD3. Moreover, AKT-mTOR signaling pathways play a key role in this regard. Metabolic changes in CD8^+^ T-cells after TLR7 stimulation include glucose regulation and glycolysis. Studies have demonstrated that glycolysis is regulated by the AKT-mTOR pathway and downstream transcription factor IRF4 (26). T-cells are dependent on diverse signals, such as T-cell antigen receptor (TCR) and proinflammatory cytokines (27, 28). CD28 is needed for activating T-cells with reduced affinity for TCR (29, 30). Some other investigations have revealed that CD4^+^ T-cells also affect TLR7 performance regulation (31, 32). However, the findings have shown that TLR7 can act as a stimulatory molecule in CD8^+^ T-cells in chronic viral infections (23).

Because of the similarity between TLR7 and TLR8, they are often described together; however there is little information regarding TLR8. TLR8 levels have been reported to be elevated in UC patients as a result of increased production of (TNF) - and IL-1 (33-35). TLR3 is involved in the detection and eradication of a variety of viruses, including coxsackie virus and herpes simplex. TLR3 recognizes dead virus-infected cells and generates IFN-1 as well as the proinflammatory cytokines TNF, IL1, and IL6, allowing the host to develop a defense against viral infections (33, 34). TLR3 has been shown to be substantially decreased in the epithelial cells of individuals with IBD (36). Respiratory syncytial virus (RSV) is the most common cause of respiratory tract infection in infants around the world. The function of TLR3 in the production of syncytial virus-induced chemokines was examined by Rudd et al. They showed that production of CCL5 by RSV was induced directly through the TLR3 signalling pathways and that there is no need for interferon signalling (IFN) by the IFN-α / β receptor (36). As a result, the TLR3 signaling pathway differs from the other TLRs. TLR3 with TLR7 play a crucial role in interferon-(IFN-)-mediated intestinal homeostasis against viruses (37). One possible mechanism for preventing intestinal inflammation by the virus involves Type I IFN signaling, which can be activated by TLR3 + 7 signaling cascades (38, 39).

**TLR-7/8 and 3 Antagonists**

TLR antagonists are immune regulators that block or reduce the activity of TLR-mediated cytokine cascades, preventing the immune system from responding. These antagonists have been investigated as potential novel treatments for autoimmune illnesses, inflammatory diseases, and cancer (40). Sainathan et al. evaluated the therapeutic effects of TLR7 antagonists on mice with IBD. Imiquimod considerably upregulated antimicrobial peptides and diminished *Salmonella typhimurium *intracellular survival. Imiquimod IFN and AMP type improved acute colitis and prevented *Salmonella* survival. Consequently, imiquimod treatments provided a new therapeutic approach for IBD patients (41). Kiyohara et al. first applied imiquimod to mice and then fed them 2% DSS (dextran sulfate sodium) in their drinking water. After that, they measured the IgM^+^ and IgD^+^ B cells of the mice. They observed a reduction in IgM^+^ and IgD^+^ B cells and an elevation in the number of macrophages, which do not release cytokines. Their results further revealed that inflammation contributed to pathogenesis through immunologic and microbiologic changes. They concluded that the antagonist of TLR7 could be a suitable option for disease treatment (42). Another investigation examined the impact of TLR7 on IL-1 as an assisting molecule for a vaccine. Overall, six chickens received inactivated IBD vaccine and others were inoculated with one dose of IBD/TIR-TLR7 vaccine. Serum antibody titer and lymphocyte proliferation were measured to estimate the humoral immunity and cellular immunity responses, respectively. The results indicated that the IBD/TIR-TLR-7 vaccine evoked specific antibody responses, while a less intense reaction was observed following the administration of inactive IBD antigen. The latter investigation demonstrated that the codelivery of TIR-TLR7 with inactivated IBD antigen led to a simultaneous augmentation in immune responses against IBD (43). In recent years, the TLRs, especially TLR7 and 9, have increasingly attracted attention. The TLR7 agonist imiquimod is applied as a local treatment for genital warts caused by the human papillomavirus (HPV) and actinic keratosis (AK). The antagonists of TLR7 and -9, such as antimalarial medications (e.g., chloroquine, hydroxychloroquine, and quinacrine), have been used since 1950 to treat immune-mediated inflammatory disorders (IMIDs), including rheumatoid arthritis, systemic lupus erythematous, and Sjogren’s syndrome. Furthermore, genetic evaluation demonstrated that TLR7 plays a fundamental role in some other IMIDs, namely multiple sclerosis (MS), IBD, and psoriasis. Consequently, ODN suppressors or small TLR7 and -9 molecules with larger safety windows and different selection might have potential clinical applications in IMID (44).

Compound 4a antagonist binds to TLR3/dsRNA to suppress TLR3 activity in gastrointestinal viral infections as well as the expression of downstream TLR3/dsRNA signaling pathways, including TNF-α and IL-1β (45). JinquanLuo et al. designed the crystal structure of the human TLR3 ectodomain (TLR3ecd) in a quadrilateral complex with three neutralizing Fab fragments that bind to the c-terminal dsRNA junction and block the interaction of TLR3ecd with dsRNA and leads to reduced production of IL-6, IL-8, MCP-1, RANTES, and IP-10 (46). Carton et al. (2012) designed a TLR3-reactive monoclonal antibody (mAb) that suppresses the secretion of IL6, IL8, and MIP1 as a result of TLR3 activation. This antibody has the potential to be utilized to treat autoimmune and inflammatory disorders (40). TRUC is a small-molecule inhibitor that competes with dsRNA binding and restricts dsRNA access to TLR3 (47). Kandimalla et al. designed oligonucleotide antagonists containing 7-deaza-dG or arabino-G to regulate the immune system in IBD. These antagonists block the signaling pathways and synthesis of a variety of cytokines, including tumor necrosis factor-alpha (TNF-), interleukin (IL) -12, IL-6, interferon (IFN) -, IL-1, and gamma-induced proteins. Moreover, they can be extremely beneficial in the treatment of diseases (48). Dynavax Technologies (DT) is a 2′-O-methyl and methoxyethyl immunosuppressive substance that can be used to suppress TLR7 and TLR8 activities in IBD individuals (52). Pharmacists have recently produced a unique CpG oligonucleotide that stimulates the immune system while acting as a TLR7 and TLR8 antagonist (40).

**Viruses and Inflammatory Bowel Disease**

The human body is a complex ecosystem comprising microbes, fungi, viruses, and other living species under the term microbiome, which usually exist on the skin and in the urogenital system. Nonetheless, the highest microbial population exists in the lower part of the digestive tract and large intestine which are involved in the digestion of food (49). Although the molecular mechanisms of inflammation of the digestive system are still unknown, studies have shown that genetics, exposure to the environment, and microbiome are among the main predisposing factors. In this regard, the development of a standard protocol for isolating, identifying, and quantifying viruses in biopsy samples of intestine and stool along with a reference scale for a healthy virus would be beneficial (50). Investigations on animal models have demonstrated that fungi and viruses can activate immune pathways. However, they can also trigger reactions that help in events related to IBD (51). Crohn’s disease and ulcerative colitis are complicated diseases which are heterogeneous in clinical, immunologic, molecular, genetic, and microbial levels. 

Although the role of intestinal viruses in IBD has been extensively studied, the data is still insufficient. The relationship between IBD, polymorphism of the FUT2 and FUT3 genes (which control the production of histo-blood group antigens), as well as rnorovirus and rotavirus ligands in the gut was documented in research in 2021 (52).

Studies on the genome have confirmed the role of autophagy in Paneth cells with disturbed cytokine secretion in the inflammatory pathways T-helper1 and T-helper17 following viral infections of the intestine. Also, the interactions between intestinal viruses and commensal bacteria can balance the viral infections of the intestine in IBD (52). Investigations into viruses for the treatment of intestinal inflammatory disease are not yet complete. The evaluation of the role viruses play using novel techniques highly assists the treatment of these diseases.

In addition to the aforementioned roles for viruses, they also activate innate immunity by TLRs, which helps to destroy the viruses. The activation of TLRs by SARS-CoV-2 leads to inflammation and IL-1β production that induces IL-6. The overactivation of inflammation in COVID-19 patients is coupled with a weak outcome (53). Therefore, TLRs play a dual role in viral infections (54-56). Moreover, TLRs causes the activation of the adaptive immune system by regulating the main series of MHCs on dendritic cells (57).

**Role of TLRs in the Pathogenesis of COVID-19**

The causative agent of COVID-19 was identified in 2019 and affected people worldwide rapidly (58). The SARS-CoV-2, as a single-stranded RNA virus, is an acute respiratory virus (SARS) from the family coronavirus (59) and is similar to viruses that caused the SARS epidemic in 2002-2004 from China and the MERS outbreak in 2012 in the Middle East. It is closely associated with the bat virus and is highly contagious. This virus is mainly spread by respiratory droplets, bats, and civets as well as pangolins which are considered as virus sources for humans (60). Although COVID-19 causes symptoms similar to influenza, it might in some cases result in severe signs, such as acute respiratory distress syndrome. It is originally a respiratory disease, although it has recently been noted to have the potential to affect multiple organs. Considering the increasing outbreak of COVID-19 in the number of people with IBD, a better understanding of the molecular mechanisms behind intestinal infections due to COVID-19 is of high importance. Patients with IBD, Crohn’s disease, and ulcerative colitis often undergo immune-suppressing treatments, such as blocking antibodies against TNF, IL-12, and IL-23, that may interfere with acute antiviral immune responses and long-lasting immune response against SARS-CoV-2 (61). The overexpression of IL-6 causes chronic inflammatory disorders, such as IBD. Research has shown that this interleukin plays a remarkable part in COVID-19, and patients with IBD are more prone to being affected by coronavirus (62, 63). These inflammatory conditions could be controlled and treated by antibody methods that target IL-6 (64). Therefore, it could be also effective in the treatment of COVID-19 (65-67). The membrane receptors of IL-6 are targeted to reduce the harmful immune signaling caused by the overexpression of IL-6 (68). As a result, there is concern that individuals with IBD are more susceptible to COVID-19 or even mortality than the general population. Furthermore, if a patient with IBD has COVID-19, there are a number of issues concerning disease management and medication interactions. As a result, learning about the mechanisms of action of viruses in IBD is critical.

Recent studies on mesenchymal stem cells (MSC) revealed that MSCs can contribute to the pathogenesis of SARS-CoV-2 by elevating anti-inflammatory cytokines and M2 macrophages (anti-inflammatory phenotype), especially TLR-7 pathways in COVID-19 patients. Several investigations have shown that TLRs play an important role in the pathogenesis of SARS-CoV (69). As mentioned above, TLRs play an important role in identifying viral particles and activating the immune system. Activation of TLR pathways leads to the secretion of inflammatory cytokines, such as IL-1, IL-6, TNF-α, and IFN-I. Diverse TLRs, including TLR-2, TLR-3, TLR-4, TLR-6, TLR-7, TLR-8, and TLR-9, are potentially important in COVID-19 (69). All the existing data concerns the pathogenesis of SARS-CoV and MERS-CoV, and studies have revealed that IFN-I plays an important role in SARS-CoV and MERS-CoV. They interfere with the signaling pathways of the host cell and reduce the expression of IFN receptors, leading to systemic inflammatory reaction (70). The production of IFN-I is regulated by TLRs and is of value in SARS-Cov and SARS-CoV-2 (71). Another investigation noted that TLR-8 is found in the lungs and contributes to the expression of cytokines, making it important in COVID-19 (72). Conti et al. confirmed that TLRs are important in COVID-19 due to their role in the production of inflammatory cytokines, such as IL-1β. Furthermore, the immunopathologic sequels in COVID-19 patients that lead to death could be attributed to the interactions between TLRs and viral particles (73). Totura et al. showed that both TRIF-based and MyD88-based pathways make the most effective antiviral defense against SARS-CoV (74). Some other studies have revealed the pathologic role of TLR-4 in hyperinflammation occurring in COVID-19 patients. As mentioned earlier, TLR-agonists can be suggested as preventive therapies for COVID-19 (75, 76). TLR-7 is a strong activator of the immune system, and the stimulation of TLR-7 leads to the release of IFN-α and TNF-α (41). Studies on variable receptors have concluded that TLR-7 causes the production of pro-inflammatory cytokines and could be regarded as a therapeutic target. According to the literature, TLRs exert both harmful and beneficial influences in COVID-19. The existing data about SARS-CoV and MERS can be useful for a better understanding of the exact role each component of the innate and adaptive immunity plays in COVID-19. Although TLR-7 and 8 are highly important in COVID-19, other receptors also need to be investigated. Moreover, the pathways related to TLRs should be assessed, because these pathways showed a relationship with mortality and sensitivity to the virus in other coronavirus families. Furthermore, bioinformatics studies can help in understanding the interactions of TLRs with proteins and COVID-19 for vaccine development.

## Conclusion

In recent years, IBD has become a major global problem due to immune system interference. As described above, examining immune-related pathways, innate immune TLR receptors, signaling pathways, and their antagonists can be very helpful in treating and preventing this disease. TLRs can be regarded as a potential target for infection control in the initial stages of the disease. In addition, the intestines are host to a variety of viruses and bacteria. Most of these viruses are bacteriophages that infect bacteria and insert genes into bacterial DNA. The close relationship between intestinal bacteriophages and bacteria increases the possible that there is a correlation between these resident viruses and IBD. It is also important to clarify the pathogenesis mechanisms of viruses to better our understanding of the key elements in IBD with the emergence of Covid-19 virus and immune system involvement. Furthermore, with the advent of the Covid-19 virus and immune system interference, a better knowledge of virus pathogenesis processes is critical for understanding the important components in IBD. The development of innate antiviral immune responses also depends on TLR3, 7, and 8. These TLRs each play an important role in IBD through different pathways affecting the immune system, and the design of TLR antagonists is very effective in IBD. As mentioned, TLR-7, 8, and 3 play important roles in the association between viruses and IBD. This research will contribute to a deeper understanding of the complex mechanism by which the virus is correlated to IBD, and it is hoped that identifying this correlation will improve the treatment of IBD patients.

## Conflict of interests

The authors declare that they have no conflict of interest.
